# Arsenic modifies the microbial community assembly of soil–root habitats in *Pteris vittata*

**DOI:** 10.1093/ismeco/ycae172

**Published:** 2024-12-27

**Authors:** Jiahui Lin, Zhongmin Dai, Mei Lei, Qian Qi, Weijun Zhou, Lena Q Ma, Randy A Dahlgren, Jianming Xu

**Affiliations:** Institute of Soil and Water Resources and Environmental Science, College of Environmental and Resource Sciences, Zhejiang University, 866 Yuhangtang Road, Hangzhou 310058, China; Zhejiang Provincial Key Laboratory of Agricultural Resources and Environment, Zhejiang University, 866 Yuhangtang Road, Hangzhou 310058, China; Institute of Soil and Water Resources and Environmental Science, College of Environmental and Resource Sciences, Zhejiang University, 866 Yuhangtang Road, Hangzhou 310058, China; Zhejiang Provincial Key Laboratory of Agricultural Resources and Environment, Zhejiang University, 866 Yuhangtang Road, Hangzhou 310058, China; The Rural Development Academy, Zhejiang University, 866 Yuhangtang Road, Hangzhou 310058, China; Institute of Geographic Sciences and Natural Resources Research, Chinese Academy of Sciences, 11A, Datun Road, Chaoyang District, Beijing 100101, China; Institute of Soil and Water Resources and Environmental Science, College of Environmental and Resource Sciences, Zhejiang University, 866 Yuhangtang Road, Hangzhou 310058, China; Zhejiang Provincial Key Laboratory of Agricultural Resources and Environment, Zhejiang University, 866 Yuhangtang Road, Hangzhou 310058, China; Department of Agronomy, College of Agriculture and Biotechnology, Zhejiang University, 866 Yuhangtang Road, Hangzhou 310058, China; Institute of Soil and Water Resources and Environmental Science, College of Environmental and Resource Sciences, Zhejiang University, 866 Yuhangtang Road, Hangzhou 310058, China; Zhejiang Provincial Key Laboratory of Agricultural Resources and Environment, Zhejiang University, 866 Yuhangtang Road, Hangzhou 310058, China; Department of Land, Air and Water Resources, University of California, One Shields Avenue, Davis 95616 CA, United States; Institute of Soil and Water Resources and Environmental Science, College of Environmental and Resource Sciences, Zhejiang University, 866 Yuhangtang Road, Hangzhou 310058, China; Zhejiang Provincial Key Laboratory of Agricultural Resources and Environment, Zhejiang University, 866 Yuhangtang Road, Hangzhou 310058, China; The Rural Development Academy, Zhejiang University, 866 Yuhangtang Road, Hangzhou 310058, China

**Keywords:** heavy metal, hyperaccumulator, homogeneous selection, core microbiome, phytoremediation

## Abstract

*Pteris vittata*, renowned for its ability to hyperaccumulate arsenic, presents a promising solution to the escalating issue of global soil arsenic contamination. This fern cultivates a unique underground microbial community to enhance its environmental adaptability. However, our understanding of the assembly process and the long-term ecological impacts of this community remains limited, hindering the development of effective soil remediation strategies. This study addresses this gap by investigating soil–root habitats from three geographically diverse fields comprising a gradient of arsenic contamination, complemented by a time-scale greenhouse experiment. Field investigations reveal that arsenic stress influences community assembly dynamics in the rhizosphere by enhancing processes of homogeneous selection. Greenhouse experiments further reveal that arsenic exposure alters the assembly trajectory of rhizosphere communities by promoting key microbial modules. Specifically, arsenic exposure increases the enrichment of a core taxon (i.e. *Rhizobiaceae*) in the rhizosphere, both in field and greenhouse settings, boosting their abundance from undetectable levels to 0.02% in the soil after phytoremediation. Notably, arsenic exposure also promotes a pathogenic group (i.e. *Spirochaetaceae*) in the rhizosphere, increasing their abundance from undetectable levels to 0.1% in the greenhouse. This raise concerns that warrant further investigation in future phytoremediation studies. Overall, this study elucidates the assembly dynamics of the soil microbiome following the introduction of a remediation plant and emphasizes the often-overlooked impacts on soil microbial community following phytoremediation. By probing the ecological impacts of remediation plants, this work advances a more nuanced understanding of the complex ecological implications inherent in phytoremediation processes.

## Introduction

The risk of arsenic pollution is increasing worldwide due to intensified industrial and agricultural activities, posing an urgent need for developing remediation technologies to reduce arsenic concentrations in soils [1]. Phytoremediation is a potentially cost-effective way to remediate highly contaminated soil by resilient plants including pioneer plants and hyperaccumulator [2, 3]. The arsenic hyperaccumulator, *Pteris vittata* (a fern species), can concentrate arsenic in its shoots up to 100-fold higher than concentrations in arsenic-contaminated environments, making it a promising candidate for the phytoremediation of arsenic-polluted soils [4, 5]. Root-associated microbial communities of *P. vittata* play an important role in helping *P. vittata* adapt to arsenic stress and enhance its arsenic uptake and accumulation. For instance, rhizosphere isolate *Cupriavidus basilensis* r507 exhibits rapid arsenite oxidation and enhances arsenic accumulation in *P. vittata* by up to 171% [6]. Another study reveals that multiple isolates from habitats associated with *P. vittata* can enhance host growth and elevate arsenic concentrations in its fronds [7]. Hence, investigating the root microbiome of *P. vittata* is crucial for advancing arsenic remediation efforts.

Ecological theory suggests that the assembly process of microbial communities associated with plants are influenced by intricate interactions involving hosts, microorganisms, and the environment [8]. On one hand, the diverse metabolic profiles of root exudates across plant species may serve as a foundation for communication and recognition, guiding the assembly and maintenance of a distinct microbiota tailored to the requirements of the host [9–11]. On the other hand, when confronted with various biotic or abiotic stresses, plants regulate gene expression linked to stress adaptation or morphological and physiological traits to recruit a “stress microbiome”, enhancing resistance to stress and safeguarding the plant [12, 13]. These ecological processes are viewed as host-driven selection for specific environmental microbiome, leading to an increased predominance of deterministic processes during community assembly.

High concentrations of metals (such as arsenic) often rank among the most significant abiotic stressors impacting plant growth and productivity [14]. However, recent studies have reported that the growth of certain plant species might benefit from nutrient acquisition services (i.e. nitrogen fixation and phosphorus solubilization) fueled by microbial arsenic oxidation [15, 16]. *P. vittata*, a typical arsenic-tolerant plant, has been recently noted for its alterations in diversity and composition of microbial community within its rhizosphere under arsenic stress [17, 18]. Furthermore, exposure to arsenic significantly altered the composition of root exudates from *P. vittata*, offering heightened selection pressure on the assembly process of rhizosphere microbial communities in response to arsenic stress [19]. However, our understanding of how arsenic stress influences the assembly process of rhizosphere microbiota in *P. vittata*, particularly across its growth and developmental phases, remains limited.

On average, 17% of the carbon fixed by photosynthesis is released by plant roots as root exudates, forming rhizodeposits that influence soil microbial communities even after the plant’s death or removal [20]. Effects of rhizodeposits are often attributed to the presence and relative balance of beneficial and pathogenic microorganisms [21]. For example, benzoxazinoid compounds and their metabolites released by maize roots alter the rhizosphere microbial community and enhance the defense capabilities of the next generation against diseases and herbivores [22]. Continuous wheat cultivation engineered a soil microbial community capable of suppressing soil-borne pathogens during successive plantings [23]. However, there remains a gap in understanding the effects of *P. vittata* rhizodeposits following phytoremediation, particularly concerning the role of arsenic in this context.

Grasping the pivotal role of arsenic in influencing microbial assembly processes in the soil–root habitats of *P. vittata*, coupled with understanding its effects on soil microbial community after phytoremediation, holds significant implications. Here, through a combination of field investigations and greenhouse pot experiments, following hypotheses were explored: (i) arsenic, rather than other abiotic factors, is the primary driver of microbial community assembly through homogeneous selection; and (ii) this selection may leave a lasting impact following phytoremediation, altering the structure of the soil microbial community. Our study found that arsenic plays a significant role in driving the assembly process of the rhizosphere community of *P. vittata*. The presence of arsenic stimulates an increase in the abundance of key rhizosphere community modules, enhances the homogeneous selection process and arsenic-associated interactions of the rhizosphere community of *P. vittata*, and ultimately produces a rhizosphere bacterial community distinct from that of ferns growing under low-arsenic conditions, leaving a lasting impact on the soil microbial community even after plant removal.

## Materials and methods

### Field sample collection

Wild *P. vittata* plants (*n* = 22) with intact roots and rhizosphere soils and adjacent bulk soils were collected from three sites in China: Zhuzhou, Hunan Province (*n* = 7, 104°31′28″-104°31′39″ E, 22°55′42″-22°56′1″ N), Daye, Hubei Province (*n* = 9, 114°48′24″-114°53′58″ E, 30°00′53″-30°04′51″ N) and Wenshan, Yunnan Province (n = 6, 121°20′38″-121°20′42″ E, 28°28′33″-28°28′40″ N). The details of sample processing methods are described in the Supporting Information (SI). Soil pH, carbon and nitrogen measurements are further detailed in the SI.

**Figure 1:**
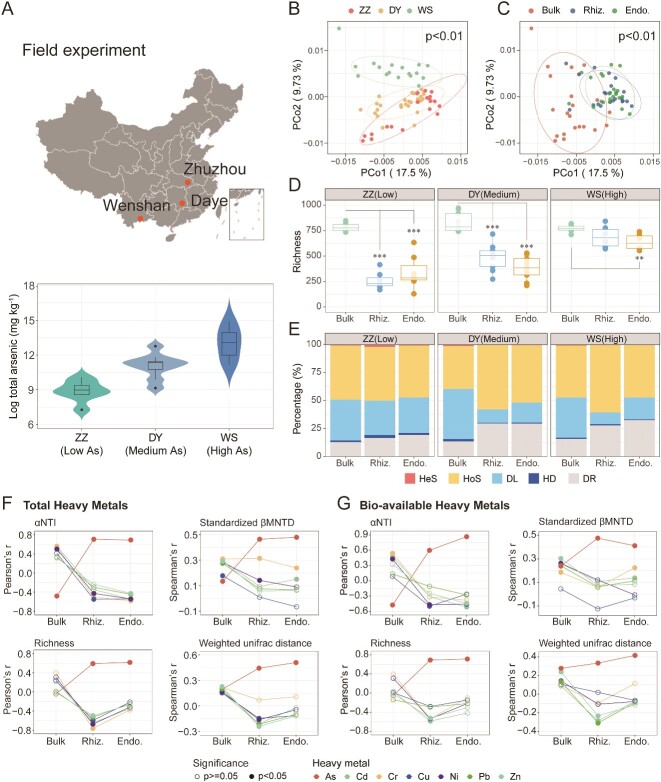
Impact of arsenic concentration on bacterial community assembly within root-associated habitats of *P. vittata* in field experiments. (A) Study site locations and total arsenic content of soil samples. (B) PCoA of bacterial community composition based on weighted UniFrac distance, separated by the three sites with different arsenic concentrations and (C) the three soil–root habitats (bulk soil, rhizosphere and endosphere); statistical significance tested using PERMANOVA. (D) Richness of three soil–root habitats for the three sites with different arsenic concentrations. The upper/lower box represent the 75th/25th quantiles, and lines within boxes indicate the median. Asterisks indicate the significance of Wilcox test (^*^: *P* < .05, ^**^: *P* < .01, ^***^: *P* < .001). (E) Quantitative inference of the relative importance of community assembly processes for the three soil–root habitats at the three sites with different arsenic concentrations by iCAMP. Colors represent heterogeneous selection (HeS), HoS, DL, homogenizing dispersal (HD), and DR, respectively. Pearson correlations between αNTI and richness with both total (F) and bioavailable (G) concentration of heavy metals (As, Cd, Zn, Pb, Cr, Cu, and Ni), and spearman correlations between βMNTD and weighted UniFrac dissimilarity with these concentrations. Solid and open symbols denote significant and non-significant correlations at *P* < .05, respectively.

Average total arsenic concentrations of Zhuzhou (low arsenic site), Daye (medium arsenic site) and Wenshan (high arsenic site) were 551, 2647, and 12 015 mg kg^−1^, respectively (Fig. 1A). Despite the gradient in arsenic levels, several important properties of the soil samples from the three sites are similar, suggesting that these indicators may have minimal impact on microbial communities (Fig. S1).

### Soil property measurements

Soil pH was measured with a pH electrode (soil:water = 1:2.5 w/v). Soil total carbon (TC) and total nitrogen (TN) concentrations (air-dried, milled <200 μm) were determined by dry combustion using an elemental analyzer (Elementar Vario EL Cube, Langenselbold, Germany). Soil dissolved organic carbon (DOC) and soil dissolved organic nitrogen (DON) were extracted by MilliQ water (soil:water =1:10 w/v), filtered through a 0.45 μm membrane filter (Millipore), and the filtrate analyzed by a Multi N/C TOC analyzer (Analytic Jena AG, Jena, Germany) [24]. Heavy metals, including arsenic (As), cadmium (Cd), zinc (Zn), lead (Pb), chromium (Cr), copper (Cu), and nickel (Ni) were measured in this study. Soil samples were placed in a 1:10 (w/v) soil-water suspension with 0.01 M CaCl_2_ to extract bioavailable heavy metals. Total heavy metals were extracted by digesting soil in a mixture of HNO_3_-HF-H_2_O_2_ (2:1:1 v/v/v) at 140°C using a microwave digester (MARS6, CEM, USA). Metal concentrations were quantified using plasma-mass spectrometry (ICP-MS; PerkinElmer Nexlon300X, Waltham, MA, USA). A certified reference soil (GBW07443) was employed for quality assurance with recovery percentages of As 95%–104%, Cd 94%–106%, Ni 81%–96%, Cr 92%–97%, Pb 92%–99%, Cu 90%–98%, and Zn 80%–92%.

### Greenhouse experiment

To verify the effect of arsenic on bacterial community assembly of soil–root habitats, the *P. vittata* separately grew in non-amended and arsenic-amended agricultural soil in a greenhouse. The pot experiment was conducted from August to September in 2023 at Zhejiang University. The soil in the experiment was obtained from a local agricultural field in June 2023 at Huzhou, Zhejiang Province (104°31′12″ E, 22°55’48″ N). The experiment consisted of four treatments: non-arsenic-amended bulk soil (control), soil amended with 80 mg kg^−1^ arsenic (arsenic-amended soil), control soil with *P. vittata*, and arsenic-amended soil with *P. vittata*. The arsenic-amended soil underwent arsenate treatment over a two-month period, with arsenic speciation measured every 20 days to confirm stabilization (Fig. S2). Uniform seedlings that had developed 8–10 leaves and measured between 6 and 7 cm were selected for transplantation and further experiments, following an approximate cultivation period of 4–6 months from the sporophyte stage. Specific details regarding cultivation of seedlings and experimental conditions are provided in SI. After transplantation, plant samples were destructively collected from each treatment every 10 days over a 30-day cultural period. Approximately 5 g soil were collected from the rhizosphere. Bulk soil sampling was performed on unplanted soil in coordination with plant collection, which included an initial sample collection and an additional sampling on the 20th day after plant removal. Each treatment included six replicates for each time point (Fig. 3A).

### Deoxyribonucleic acid extraction and sequencing

Soil deoxyribonucleic acid (DNA) was extracted using the MP FastDNA SPIN Kit (MP Biomedicals, USA) following manufacturer’s instructions. DNA purity and quantity were determined using a Nano-Drop spectrophotometer (NanoDrop Technologies, USA). Extracted DNA was stored at −80°C. The sequencing of the V4-V5 region of the 16S ribosomal ribonucleic acid (rRNA) gene was conducted using primers 515F and 907R on a NovaSeq 6000 platform (Illumina, USA), resulting in ~6.5 million paired-end reads with a length of 250 bp.

### Bioinformatic analysis

Sequence quality assessment was performed using FastQC [25], establishing low-quality cutoffs for both forward and reverse readings. The QIIME 2 (2020.2v) pipeline [26] was employed to process the data. The cutadapt plugin was utilized to remove primers. The “dada2” denoise-paired plugin was used for filtering and trimming of low-quality reads (−-p-trunc-len-f 0, −-p-trunc-len-r 244), error correction by modeling expected error rates, dereplication, and chimera removal to yield accurate amplicon sequence variants (ASVs). For taxonomic assignment, a naive Bayes classifier was trained with the Silva 138 Reference Taxonomy Database (https://www.arb-silva.de/download/archive/). The q2-feature classifier plugin was employed to assign taxonomy to sequences, using a confidence threshold of 0.8 to acquire the ASV information.

### Microbial diversity

Microbial alpha diversity was performed using R package “vegan”. Beta diversity (weighted UniFrac distances) analysis was performed with R package “GUniFrac”. The unconstrained principal coordinates analysis (PCoA) was performed on Bray-Curtis distances to quantify the beta diversity.

### Microbial assembly process

Analysis of phylogenetic signal based on total arsenic content identified a significant correlation between arsenic niche differentiation and phylogenetic distance among species, observable within a defined threshold (0.13; Fig. S3). The community assembly process was assessed by using pNST (rand.time = 1000) [27] and iCAMP (ds = 0.13, bin.size.limit = 24, rand.time = 1000, phylo.rand.scale = “within.bin”, taxa.rand.scale = “across.all”, phylo.metric = “bMPD”) [28]. To further evaluate the phylogenetic clustering and community turnover rates, NTI and βMNTD (normalized to a range of 0 to 1) indices were extracted from the calculated metrics.

### Co-occurrence networks

Bacterial co-occurrence networks were constructed based on Pearson correlations of log-transformed ASV abundances and heavy metal concentrations, followed by an random matrix theory (RMT)-based approach that determines the correlation cut-off threshold in an automatic fashion. Indirect correlation dependencies were distinguished using the network enhancement method to restore the real co-occurrence relationship [29]. The Molecular Ecological Network Analysis Pipeline (MENAP) was used for network construction (http://ieg4.rccc.ou.edu/MENA/) [30]. R Package “igraph” was used to calculate network topology parameters, and identify heavy metal correlated modules. Gephi software was applied to visualize co-occurrence networks.

### Statistical analysis

Kruskal–Wallis test was used to evaluate differences in soil properties among sites categorized according to low, medium, and high arsenic concentrations. ANOSIM test in R package “vegan” was used to assess differences between the compositions of communities. A t-test was employed to assess differences in sample positions along principal coordinate axis and differences in relative abundances. The “DESeq2” R package [31] was used to implement negative binomial generalized models to assess the effect of experimental factors on the abundance of individual ASVs. Pairwise Wald tests were used for individual difference testing. The Benjamini–Hochberg procedure was used to adjust P values. All plots were generated with “ggplot2” R package.

## Results

### Microbial assembly features in arsenic-contaminated soil–root habitats of *Pteris vittata*

Bulk soils corresponding to *P. vittata* from the three sites share similar soil properties (Fig. S1 and Table S1), but show an increasing gradient of arsenic concentration (Fig. 1A, Table S2, and Table S3). Consequently, soil–root samples are classified as low, medium, and high arsenic-contaminated groups according to site.

In terms of beta-diversity, the community composition of bulk soils from the three sites is clearly distinct. However, the corresponding *P. vittata* root-associated habitats exhibit higher similarity, highlighting the strong filtering effects of plant roots on microorganisms (Fig. 1B and C). Regarding alpha-diversity, the microbial richness of bulk soil is comparable across the three sites. Interestingly, while microbial diversity in root-associated habitats decreases compared to bulk soil, it increases as arsenic contamination escalates (Fig. 1D). The importance of inference on community assembly processes suggests that homogeneous selection (HoS), dispersal limitation (DL), and “drift” (DR) are the primary assembly processes in soil–root habitats of *P. vittata*. Additionally, the results revealed stronger homogeneous selection in both the rhizosphere (HoS: 54.9%) and endosphere (HoS: 45.9%) compared to bulk soil (HoS: 41.6%) and an escalating homogeneous selection process in rhizosphere from the low-to-high arsenic contaminated sites (Fig. 1E). Notably, multiple linear regression analysis reveals that arsenic has the most significant impact on rhizosphere microbial richness (Table S4). Redundancy analysis further emphasizes that total arsenic and bioavailable arsenic, rather than other environmental factors, are the primary drivers shaping the composition of the rhizosphere microbial community (Fig. S4).

**Figure 2:**
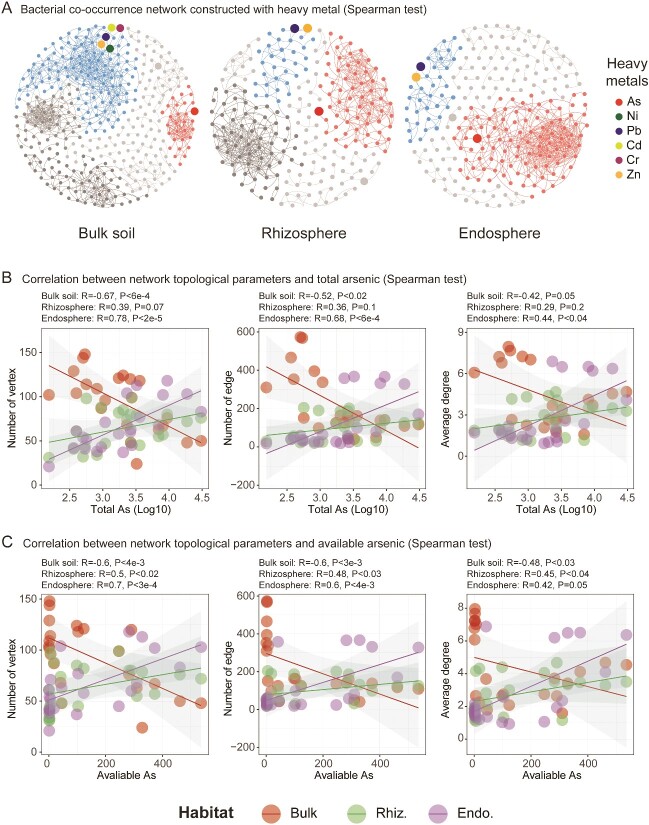
Increased complexity within arsenic-associated network modules in soil–root habitats correlates with rising arsenic concentration. Overall bacterial co-occurrence networks (A) in the bulk soil, rhizosphere, and endosphere. Node size reflects the relative abundance of each bacterial taxa, except for the heavy metal elements (As, Ni, Pb, Cd, Cr, Zn) related to modules, which are denoted by larger nodes. Modules in the overall networks represent arsenic-regulated and other heavy metal-regulated bacterial gatherings. Spearman’s correlations between total (B) or bioavailable (C) arsenic concentrations and the node number, edge number, and average degree in bacterial co-occurrence network of bulk soil, rhizosphere, and endosphere.

Moreover, the total/bioavailable concentrations of arsenic influenced the interactions within the microbial community in soil–root habitats. The topological parameters of the co-occurrence network (i.e. node number, edge number, and average node degree) were positively correlated with total/available arsenic concentrations in root-associated habitats, whereas an opposite trend was observed in bulk soil (Fig. 2B and C, Spearman test). Importantly, the network module linked to arsenic was distinctly separated from modules associated with other heavy metals, and the complexity of arsenic-linked modules increased from bulk soil to rhizosphere to endosphere (Fig. 2A, Table S5). Further investigation of arsenic-related microbial modules revealed a significant presence of bacteria from the Rhizobiales order. In the rhizosphere, a considerable number of nodes belonged to the *Rhizobiaceae* family, including bacteria from the genera *Ensifer*, *Rhizobium*, and *Mesorhizobium* (Fig. S5).

Correlation analysis further supported significant positive relationships between total/bioavailable arsenic concentration and community α-diversity (richness), β-diversity (weighted UniFrac dissimilarity), assembly (α-NTI and standardized β-MNTD), and interactions (nodes, edges and degrees) in root-associated habitats, which are distinctly different from those observed in bulk soil, or with other heavy metals (e.g. Cd, Cr, Cu, Ni, Pb, and Zn; Fig. 1F and G, Fig. 2B and C) or nutrient elements (e.g. pH, DOC, TC, DON, and TN; Fig. S6).

In summary, arsenic plays a unique role in shaping the turnover of microbial communities within the root-associated habitats of *P. vittata*, particularly in the rhizosphere. This leads to increased alpha diversity, enhanced homogeneous selection processes, and strengthened arsenic-related microbial interactions.

### Arsenic exposure modifies compositional development of rhizosphere microbial community

**Figure 3:**
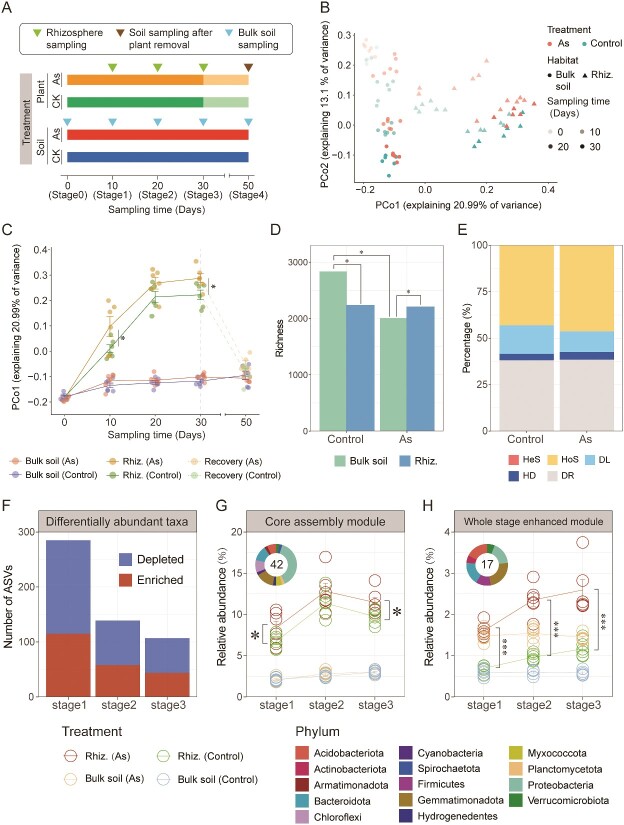
Rhizosphere community dynamics and ASVs responsive phylum to host selection under arsenic stress. (A) Timeline of experimental sample collection for control and arsenic-treated plants and bulk soil. Inverted triangles mark each sampling time point, three for rhizospheric sample collection, five for bulk soil, and one for soil after plant removal. (B) PCoA of bacterial community composition of bulk and rhizosphere soil with and without arsenic exposure based on Bray–Curtis distance. Principal coordinate 1 (PCo1) represents the direction of greatest variation among samples, while principal coordinate 2 (PCo2) captures additional distinct information from PCo1, indicating the second direction of greatest variation. (C) Developmental trend of microbial community in the first principal component over time. The trend lines represent shifts in mean values, with statistical significance determined by the Wilcoxon test. (D) Richness of bulk and rhizosphere soil with and without arsenic exposure at developmental stage 3 (30 days). (E) Quantitative inference of the relative importance of community assembly processes in the rhizosphere with and without arsenic exposure at developmental stage 3 (30 days) by iCAMP. (F) Number of differentially abundant ASVs (*P* < .05, FDR < 0.1) detected in rhizosphere with and without arsenic exposure at three developmental stages. Statistical significance was determined by negative binomial generalized linear models and pairwise Wald tests (two-sided) corrected with the Benjamini–Hochberg procedure. Temporal trend for the relative abundances of CAM (G) and whole stage enhanced module (H) in bulk and rhizosphere soil with and without arsenic exposure across three developmental stages. Trend lines represent the shifts of mean values and inset donut plots display the proportion (number of ASVs) and taxonomic composition of each module. Asterisks indicate the significance of Wilcox test (^*^: *P* < .05, ^**^: *P* < .01, ^***^: *P* < .001).

To further understand the impact of arsenic on community assembly within the root-associated habitats of *P. vittata*, a greenhouse experiment was conducted to characterize the assembly dynamics of rhizosphere microbial communities across three developmental stages. Additionally, soil samples with and without arsenic exposure 20 days after removing the plants were collected to characterize the effects of arsenic and plant presence on soil microbiome (Fig. 3A). PCoA depicted the compositional changes of microbial communities over time (Table S6). Principal Coordinate 1 (PCo1), indicating the direction of greatest variation between samples, demonstrates a gradual separation of rhizosphere community structures from bulk soils across early to late developmental stages along this axis. Meanwhile, Principal Coordinate 2 (PCo2) captures distinct additional information from PC1, representing the second direction of greatest variation and revealing consistent variation patterns over time in both bulk and rhizosphere soils (Fig. 3B). Temporal dynamics analysis of PCo1 demonstrated similar developmental trajectories for rhizosphere communities with and without arsenic exposure, ultimately stabilizing at stage 3. However, rhizosphere communities with arsenic exposure consistently diverged from those without arsenic exposure from stage 1 to stage 3, forming a distinct community structure. In contrast, bulk soils showed no significant differences over time or with arsenic treatment (Fig. 3C).

To validate the field investigation findings, the assembly features of microbial communities at developmental stage 3 were analyzed. The results indicate that in the control group, the rhizosphere filtering effect significantly decreased bacterial richness compared to bulk soil. In contrast, under arsenic exposure, rhizosphere richness was significantly greater than that of bulk soil. Although arsenic exposure substantially reduced bacterial richness in bulk soil, complicating direct comparisons with rhizosphere communities, this observation indirectly suggests an enhancement of bacterial richness linked to arsenic (Fig. 3D). Additionally, arsenic treatment increased the relative importance of homogeneous selection in community assembly (Fig. 3E). These findings support the conclusions from the field investigation.

To deeper investigate the impact of arsenic exposure on community assembly, negative binomial models were used to identify differentially abundant ASVs. Pairwise Wald tests were conducted to compare rhizosphere communities exposed to arsenic with those not exposed and with the corresponding bulk soil at each developmental stage. A total of 355 ASVs were found to be affected by treatment in at least one comparison (Fig. S7, *P* < .05, P_FDR_ < 0.1). The number of differentially abundant ASVs gradually decreased and stabilized as rhizosphere community assembly progressed (Fig. 3F). Notably, significant differences in the community structure between arsenic-exposed and control-group rhizospheres persisted, even as the number of differentially abundant ASVs diminished.

Two microbial modules identified from differentially abundant ASVs were significant contributors to increased homogeneous selection in the rhizosphere under arsenic exposure: the core assembly module (CAM) and whole stage enhanced module (WSEM; Table S7 and Table S8). The CAM, defined as the ASVs present in each rhizosphere sample and significantly enriched compared with bulk soil from the first stage to the third stage, constituted 42 ASVs and represented ~10% of the rhizospheric community’s relative abundance. This was four times higher than its proportion in non-rhizosphere soil (2.6%). This module exhibited a stable rhizosphere enrichment effect throughout the entire growth period, primarily composed of microorganisms from Proteobacteria (38%), Gemmatimonadota (14.3%), and Bacteroidota (12%; Fig. 3G). The WSEM, defined as ASVs present in each arsenic-treated rhizosphere samples and significantly enhanced compared with the control-group rhizosphere across all stages, consisted of 17 ASVs and was significantly enhanced in the rhizosphere community under arsenic treatment. They comprised an enrichment level of ~2.2%, which was 2.4 times higher than the control group’s rhizosphere community. Abundances within this module for the rhizosphere increased gradually with plant growth, both in arsenic-treated and control groups, with a direct response to arsenic addition, stabilizing at a ~ 2.5-fold increase. This module was primarily composed of Gemmatimonadota (23.5%), Proteobacteria (17.6%), Bacteroidota (17.6%), and Acidobacteriota (17.6%; Fig. 3H).

Overall, the greenhouse experiment traced the development of rhizosphere communities, confirming the field investigation findings and providing further insights into the reasons for arsenic-enhanced homogeneous selection in rhizosphere community assembly.

### Impact of arsenic on microbial assembly processes in the rhizosphere and soil microbes following phytoremediation

**Figure 4:**
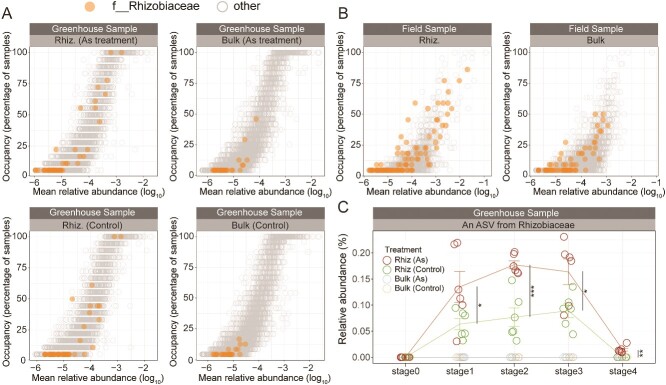
Occupancy–abundance relationship and abundance dynamics of *Rhizobiaceae* taxa in the greenhouse experiment. (A) Occupancy–abundance relationships for the rhizosphere and bulk soil communities with and without arsenic exposure in greenhouse experiments. (B) Occupancy–abundance relationships for bulk and rhizosphere soil in field samples. The x-axis displays the log-transformed mean relative abundance for each ASV, while the y-axis displays the percentage of samples in which each ASV was detected. ASVs from *Rhizobiaceae* points are colored orange. (C) Shifts in the cumulative relative abundance of *Rhizobiaceae* and a specific member of *Rhizobiaceae* throughout the entire process of community assembly. Trend lines represent the mean values for each treatment throughout the experiment. Asterisks indicate the significance of Wilcox test (^*^: *P* < .05, ^**^: *P* < .01, ^***^: *P* < .001).

Further exploration focused on the enrichment effects on specific taxa using abundance change curves. A notably enriched ASV (e.g. ASV1) from the *Rhizobiaceae* family was identified in both CAM and WSEM. Under arsenic stress, the average relative abundance of ASV1 increased from nearly undetectable levels (<10^−5^) in bulk soil to ~0.1% in the rhizosphere community by stage 3. Furthermore, arsenic treatment significantly enhanced the abundance of this taxon in the rhizosphere to an average of 0.16% at stage 3. Abundance change curves over time indicate that ASV1 was assembled in the early developmental stage of the rhizosphere community, showing a gradual increase with plant growth and development (Fig. 4C). Without arsenic exposure, the abundance of ASV1 in the soil dropped sharply to undetectable levels 20 days after plant removal. However, with arsenic exposure, the resident abundance of ASV1 in soil increased to 0.02%. Moreover, the abundance–occupancy curves from both field and greenhouse experiments indicate a stable and broad-spectrum enrichment effect of the *Rhizobiaceae* family in the rhizosphere of *P. vittata* (Fig. 4A and B). Specifically, the rhizosphere exhibited a higher number of species with both high abundance and high occupancy within the *Rhizobiaceae* family compared to the corresponding bulk soils. This phenomenon was particularly pronounced under arsenic exposure.

**Figure 5:**
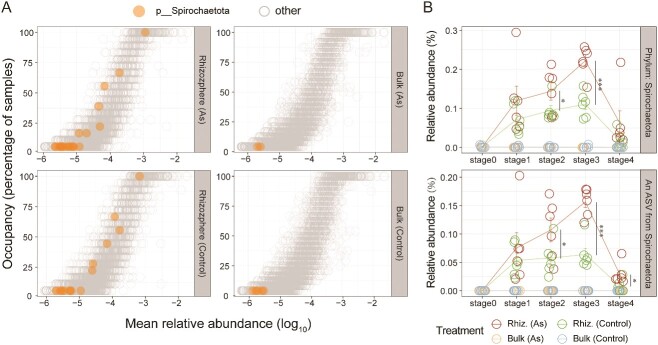
Occupancy–abundance relationship and abundance dynamics of Spirochaetota taxa in the greenhouse experiment. (A) Occupancy–abundance relationships for the rhizosphere and bulk soil communities with and without arsenic exposure in greenhouse experiments. (B) Shifts in the cumulative relative abundance of Spirochaetota and an ASV from Spirochaetota throughout the entire process of community assembly for bulk and rhizosphere soils with and without arsenic treatment. Trend lines represent the mean values for each treatment throughout the experiment. Asterisks indicate the significance of Wilcox test (^*^: *P* < .05, ^**^: *P* < .01, ^***^: *P* < .001).

Our exploration extended to the enrichment effects of rare soil taxa, revealing significant enrichment of the Spirochaetota phylum, which is typically rare in bulk soil (Fig. 5A). Notably, a taxon from the *Spirochaetaceae* family (ASV2) was detected in CAM, showing a substantial increase in abundance from undetectable levels in bulk soil to ~0.1% in the rhizosphere community (Fig. 5A and B). Temporal dynamics indicated an increased abundance, peaking at ~0.16%, under arsenic exposure across all developmental stages (Fig. 5B). After plant removal, the abundance of Spirochaetota in the arsenic-exposed soil increased compared to the bulk soil without arsenic exposure (*P* = .05). The abundance of ASV2 significantly increased with arsenic exposure compared to both bulk soil (*P* < .01) and the control (*P* < .05).

In total, arsenic is shown to enhance the rhizosphere enrichment of specific core microorganisms, notably *Rhizobiaceae*, subsequently increasing their soil abundance following phytoremediation. Furthermore, arsenic may also elevate the abundance of rare microbes in the rhizosphere, such as *Spirochaetaceae*, potentially increasing the risk of soil-borne pathogens.

## Discussion

### *Pteris vittata* “Cry for Help” under increased arsenic stress

The commonly observed loss in bacterial diversity from soil to rhizosphere to endosphere indicates plant-driven selection of microbiome at the root-soil interfaces [8]. In field experiment, our results consistently showed a decrease in bacterial richness within root-associated microbial communities of *P. vittata* compared to bulk soil, further indicating a root-mediated selection process. Despite significant differences between microbial communities in bulk soil from the three geographical locations, the bacterial community within the root-associated habitats of *P. vittata* is more homogeneous (Fig. 1B, C, and E). Interestingly, although diversity in the rhizosphere and endosphere decreases compared to bulk soil, it increases with rising arsenic concentrations (Fig. 1D). Our greenhouse experiments lend further support to this conclusion. Despite arsenic exposure markedly reduces bacterial diversity in bulk soil, promoting a higher diversity in rhizosphere community relative to the corresponding bulk soil (Fig. 3D). This effect likely results from several potential mechanisms: (i) Compared to bulk soil with high arsenic concentration, the substantial uptake of arsenic by *P. vittata* may reduce the toxicity in the rhizosphere [4]. (ii) Arsenic exposure uniquely induces the release of diverse metabolites from the roots, enhancing the metabolism, detoxification, and signaling functions of soil microorganisms, thereby fostering greater biodiversity and microbial interactions. (iii) Arsenic exposure drives the efflux of As(III), fueling arsenic oxidation in the rhizosphere and supplying electron donors to the microenvironment [7, 32]. This process may support a cascade of biochemical activities, such as nitrogen fixation and phosphorus solubilization [15].

The unique and robust correlations between arsenic concentration and measures of richness, α-NTI, and β-MNTD indicate that increased arsenic stress not only enhances species diversity but also aligns with the turnover of rhizosphere microbial communities, leading to stronger phylogenetic clustering toward terminal branches (Fig. 1F and G). This could result from the unique “cry for help” mechanism in *P. vittata* by arsenic stress. Recent studies have unveiled that plants can assemble beneficial rhizomicrobiomes through a “cry for help” mechanism to combat biotic and abiotic stress in terrestrial ecosystems. For example, maize produces apigenin and luteolin under nitrogen deprivation, promoting the enrichment of *Oxalobacteraceae* bacteria in the rhizosphere, which enhances plant growth and nitrogen acquisition by stimulating root proliferation [33]. Fusaric acid produced by *Fusarium oxysporum* mediates the assembly of disease-suppressive rhizosphere microbiota via induced shifts in root exudates of tomato plants [34]. Metabolomics studies have shown that arsenic exposure induces a significant shift in the metabolite profile of *P. vittata* [19]. Additionally, metabolites secreted by *P. vittata* roots play a crucial role in shaping the rhizospheric microbial community, which enhances arsenic extraction efficiency and promotes the growth of the plant [17]. These findings suggest that environmental arsenic stress, as opposed to other abiotic factors, may activate a distinct metabolite regulation mechanism that drives the selective assembly of specific microbial groups in the rhizosphere.

### Key rhizosphere microbes responding to arsenic stress

Our greenhouse experiments revealed changes in the rhizospheric microbiome of *P. vittata* during its growth and development, as well as the impact of arsenic stress on this process. Turnover of microbial communities in the early stages of plant growth and development was higher, gradually decreasing to a stable level ~5 weeks after transplant (Fig. 3C). This trend is similar to the development of rhizosphere communities in other plant life cycles. For instance, the rhizosphere community in rice stabilizes ~8–9 weeks after germination [35]. Interestingly, arsenic exposure was accompanied by development of the whole rhizosphere community. This indicates that arsenic regulates the assembly process of the rhizosphere community of *P. vittata* throughout its life cycle.

Further clustering of ASVs based on abundance differences between arsenic exposure and control groups revealed multiple modules that enhance deterministic processes in community assembly. The enhancement of CAM occurs early in plant growth and development, persisting throughout development of the rhizosphere community (Fig. 3G). This assembly pattern may be related to the recently proposed concept of a “core microbiome” within plants—a subset of the plant microbiome consistently associated with specific crop species across a broad spatial scale [8]. Regardless of environmental conditions, these core microorganisms maintain persistent relationships with the host, encompassing key microbial taxa carrying functional genes crucial for the host’s health. Species within the CAM constitute ~0.05% of the total detected species in the community, yet they exhibit high relative abundance (~10% of community). This includes bacteria from *Sphingomonas*, potentially being one of the major arsenic-reducing groups in root-associated habitats [36]. It also includes some bacteria from *Rhizobiaceae*, recognized as a key arsenic-oxidizing group within *P. vittata* roots [37]. Remarkably, a rhizosphere-isolated *Rhizobiaceae* strain demonstrates strong arsenic-oxidizing activity, driving more arsenic accumulation in roots and fronds while enhancing plant growth [7, 38]. This effect may be due to arsenic oxidation effectively increasing the levels of As(V) in the rhizosphere, thereby enhancing the arsenic uptake efficiency of *P. vittata* [4, 39]. Moreover, several known members of *Rhizobiaceae* positively impact plant growth through production of growth-regulating hormones, nutrient mobilization, and/or protection of plants from biotic and abiotic stressors [8]. The activation of this module is possibly responsible for *P. vittata*’s resilience against heavy metal stress and growth constraints [40].

The WSEM experiences dual selection pressures from both arsenic and roots. Prior to transplantation, bacteria from this module were consistently enriched in bulk soils when exposed to arsenic (Fig. 3H). This phenomenon may stem from metabolic disparities within the microbial community. For instance, arsenic toxicity inhibits the growth of some arsenic-sensitive microbes in soil, thereby creating niches for the growth of other arsenic-tolerant microbes [41]. After transplantation, the emergence of rhizosphere exudates further selects for microorganisms, expanding the niche for arsenic-adapted and metabolically synchronized taxa. This module encompasses numerous bacterial genera known to be associated with associated with arsenic dynamics. For instance, strain from *Gemmatimonas* and *Lysobacter* exhibit high levels of arsenic tolerance and are frequently present in arsenic-contaminated environments [42–44]. Strains from *Opitutus* are identified for their arsenic-reducing capabilities [45, 46], while those from *Pseudomonas* have shown potential for lead immobilization, thereby reducing lead uptake and promoting the growth of *P. vittata* [47]. This evidence suggests that the arsenic-regulated rhizosphere bacterial module WSEM may facilitate arsenic transformation and uptake by *P. vittata*, while aiding in plant resilience to other heavy metal stresses.

### Impact of phytoremediation on soil microbial community

Soil microorganisms are both resistant and resilient [48]. Certain microbial groups show significant metabolic flexibility and physiological tolerance to changing environmental conditions [49], enabling them to occupy niches that are resistant to change. Additionally, traits such as widespread dispersal and rapid growth suggest that microbial communities can quickly recover from disturbances [50]. These ideas could account for the recovery of the soil microbiome, which generally returns to resemble that of bulk soil after plant removal (Fig. 4C). However, a significant difference was observed in the overall microbial community between the recovery soil and bulk soil under arsenic exposure (ANOSIM for arsenic exposure treatment: R = 0.5, *P* = .01; ANOSIM for control: R = −0.01, *P* = .4). This highlights the more persistent changes in the soil microbiome induced by *P. vittata* under arsenic exposure.

Importantly, we observed that arsenic exposure promotes a persistent increase in the abundance of previously rare microbes in the soil. Members of Rhizobiales are a part of the core microbiome and the dominant arsenic oxidizers in *P. vittata* roots [32]. Our field investigations revealed that taxa from *Rhizobiaceae*, a family within Rhizobiales, are broadly enriched in the rhizosphere of *P. vittata* (Fig. 4B). The greenhouse experiments furtherly showed that a more significant enrichment of *Rhizobiaceae* taxa when exposed to arsenic stress (Fig. 4A). The abundance variation curve demonstrates that arsenic exposure leads to a significant increase in the abundance of a *Rhizobiaceae* taxon throughout the entire developmental process of the rhizosphere microbiome in *P. vittata* (Fig. 4C). This suggests its important role in the rhizosphere of *P. vittata* in response to arsenic stress. Notably, arsenic exposure resulted in a significantly higher abundance (0.02%) of this taxon in the soil 20 days after plant removal compared to the bulk soil. Several studies have reported the role of *Rhizobiaceae* in nitrogen fixation, phosphorus solubilization, and stress resistance, indicating its potential as a beneficial microbial group for improving soil functions and promoting plant growth [51–53].

In addition to the positive effects associated with the accumulation of beneficial or symbiotic microorganisms, plants can also exert negative effects on subsequent plant species by promoting the accumulation of pathogenic microorganisms in the soil [54]. Greenhouse experiments revealed a notable enrichment of the Spirochaetota phylum in the rhizosphere (Fig. 5A). The abundance variation curve demonstrates that arsenic exposure significantly enhances the abundance of this phylum during the later stages of rhizosphere community development (Fig. 5B). One ASV from *Spirochaetaceae* notably contributes to this enrichment, exhibiting a significantly increased abundance (0.1%) in the soil after plant removal. Some species within *Spirochaetaceae* are known to cause human diseases, including syphilis, Lyme disease, and epidemic and endemic forms of relapsing fever [55]. While this phenomenon was not detected in our field investigations, the possibility of pathogenic microorganisms being enriched in the soil after phytoremediation cannot be disregarded.

## Conclusions

Our findings reveal that arsenic stress uniquely affects microbial community assembly in the rhizosphere of the As-hyperaccumulator *P. vittata*, enhancing homogeneous selection for core taxa and mediating distinct impacts on the soil microbial community after phytoremediation. Crucially, although this phenomenon was not observed in field conditions, the enrichment of pathogenic groups (*Spirochaetaceae*) during and after phytoremediation in the greenhouse setting highlight concerns that merit closer attention in future phytoremediation research. These findings, however, provide only an initial insight of the impacts of soil microbiome by phytoremediation processes. The deployment of remediation plant species from diverse plant phyla could drive a spectrum of soil ecological responses. Consequently, a more thorough investigation into the assembly of rhizosphere microbial communities across diverse remediation species their effects on the soil microbial community after plant removal could yield a deeper understanding of the ecological dimensions of phytoremediation, providing crucial insights for selecting plant–microbe combinations that optimize remediation strategies. Moreover, understanding the persistence of impacts on soil microbial community following phytoremediation is essential for identifying safer species and optimizing remediation strategies. This study provided a preliminary assessment of the impacts of microbial community assembly in *P. vittata* on the soil microbiome over a 20-day period, and future research should expand this timeframe to capture longer-term dynamics.

## Supplementary Material

Revised_Supplementary_Information_ycae172

## Data Availability

The DNA sequencing data are available at the National Center for Biotechnology Information under the project accession PRJNA1045988.
