# Supplementary material for: Arsenic modifies the microbial community assembly of soil–root habitats in Pteris vittata
Source: ISME Commun. 2024 Dec 27;5(1):ycae172. doi: 10.1093/ismeco/ycae172 (PMC11742257; doi:10.1093/ismeco/ycae172)
Supplement: Revised_Supplementary_Information_ycae172 [file revised_supplementary_information_ycae172.pdf]

Supplementary Information

Arsenic modifies the microbial community assembly of soil-root habitats in *Pteris vittata*

Running title: Microbial response to As in fern soils

Jiahui Lin[a,b #], Zhongmin Dai[a,b,c #], Mei Lei[d], Qian Qi[a,b], Weijun Zhou[e], Lena Q. Ma[a,b], Randy A. Dahlgren[f], Jianming Xu[a,b,c*]

[a] Institute of Soil and Water Resources and Environmental Science, College of Environmental and Resource Sciences, Zhejiang University, 866 Yuhangtang Road, Hangzhou 310058, China

[b] Zhejiang Provincial Key Laboratory of Agricultural Resources and Environment, Zhejiang University, 866 Yuhangtang Road, Hangzhou 310058, China

[c] The Rural Development Academy, Zhejiang University, Hangzhou 310058, China

[d] Institute of Geographic Sciences and Natural Resources Research, Chinese Academy of Sciences, Beijing 100101, China

[e] Department of Agronomy, College of Agriculture & Biotechnology, Zhejiang University, Hangzhou 310058, China

[f] Department of Land, Air and Water Resources, University of California, Davis, 95616 CA, USA

***Corresponding author:** Jianming Xu, Institute of Soil and Water Resources and Environmental Science, College of Environmental and Resource Sciences, Zhejiang University, 866 Yuhangtang Road, Hangzhou, Zhejiang, 310058, China.

# Jiahui Lin and Zhongmin Dai contributed equally to this work.

Sample collection and processing

Bulk soil was collected ~5 cm from plant roots to avoid the influence of root processes on the soil material. To minimize the impact of experimental procedures on the results, the bulk soil was subsequently processed in the same way as the rhizosphere soil. The rhizosphere soil was composed of soil extending approximately 1-3 mm from the root tissue. The rhizosphere soil collection method was comprised of gently shaking roots to remove loosely bound soil, then placing the roots in a 25 mL phosphate-buffered saline (PBS) solution, which was vortexed twice for 30 s each time. After removing the roots, the suspension was centrifuge at 8000 *g* for 5 min, supernatant discarded, and the sediment collected as rhizosphere soil. Endosphere materials were obtain by placing the rhizosphere-extracted roots in a centrifuge tube containing 25 mL PBS buffer and vortex oscillated for 15 s with subsequent disposal of the suspension. This step was repeated until the PBS buffer was completely clarified. Roots were then ultrasonicated at a frequency of 50-60 Hz for 30 s. After 5 sonication cycles, the suspension was eliminated, and the roots were sterilized and freeze-dried at -50℃. The freeze-dried root tissues were ground and the powders collected to isolate the endosphere microbial community. All the samples were stored at -80℃ prior to further analyses.

Cultivation of *Pteris vittata* seedlings

Pteris vittata (L.) spores were subjected to surface sterilization by sequential immersion in 75% ethanol for 2 min and 10% sodium hypochlorite for 12 min, followed by rinsing in sterile Milli-Q water. The sterilized spores were suspended in 2 mL sterile Milli-Q water and uniformly dispersed. The spores were germinated on Petri dishes containing Murashige and Skoog solid medium. Petri dishes were placed in a growth chamber under warm fluorescent lamps with a 14 h photoperiod, a light intensity of 180 mmol m^{-2} s^{-1}, 60% humidity, and a day/night temperature of ~26 ℃/20 ℃. After ~20 d, spores germinated and gametophytes were subcultured into fresh media monthly. After 70 days of cultivation, sporophytes emerged and were subcultured into fresh media bimonthly. After three transfers, uniform seedlings that had developed 8-10

leaves and measured between 6-7 cm were selected for transplantation and further experiments.

Experimental conditions for pot plants in greenhouse

The entire experimental process took place in a greenhouse with constant temperature (28°C), humidity (70%), and light conditions (12 hours of light and 12 hours of darkness). Arsenate solution (sodium arsenate dibasic heptahydrate; Sigma-Aldrich, Missouri, USA) was uniformly added to an agricultural soil to achieve a total arsenic content of 80 mg kg^{-1}, and the soil moisture level adjusted to 60% of total water holding capacity. The control soil was treated with water, but no As, in an otherwise identical manner.

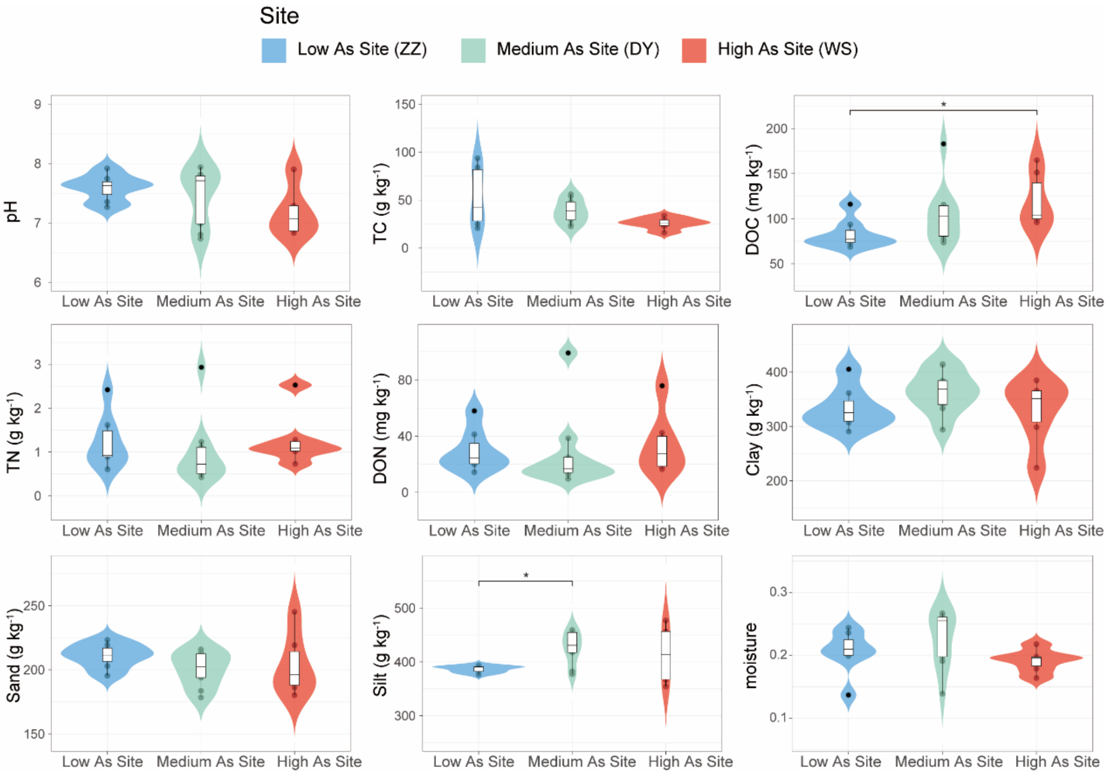

Figure S1 Violin plots illustrate the data distribution of basic soil properties including dissolved organic nitrogen (DON), total nitrogen (TN), pH, dissolved organic carbon (DOC), total carbon (TC), sand, silt, clay and moisture across low-, medium-, and high-As sites. Inside the violin, a box plot typically shows the median (center line), interquartile range (box edges), and possibly outliers (dots or points beyond the whiskers). The Kruskal-Wallis test was used to assess differences among groups, followed by pairwise Wilcoxon tests for comparisons. Asterisks denote statistical significance (*: $p < 0.05$).

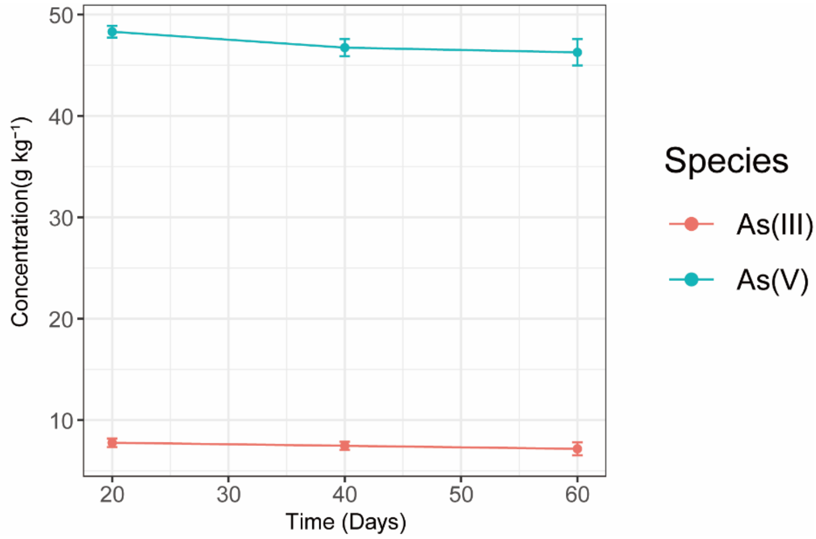

Figure S2 The line chart shows the trend of concentration changes over time for As(III) and As(V) in the soil after As(V) was added. Data are shown as mean ± SEM (n = 3).

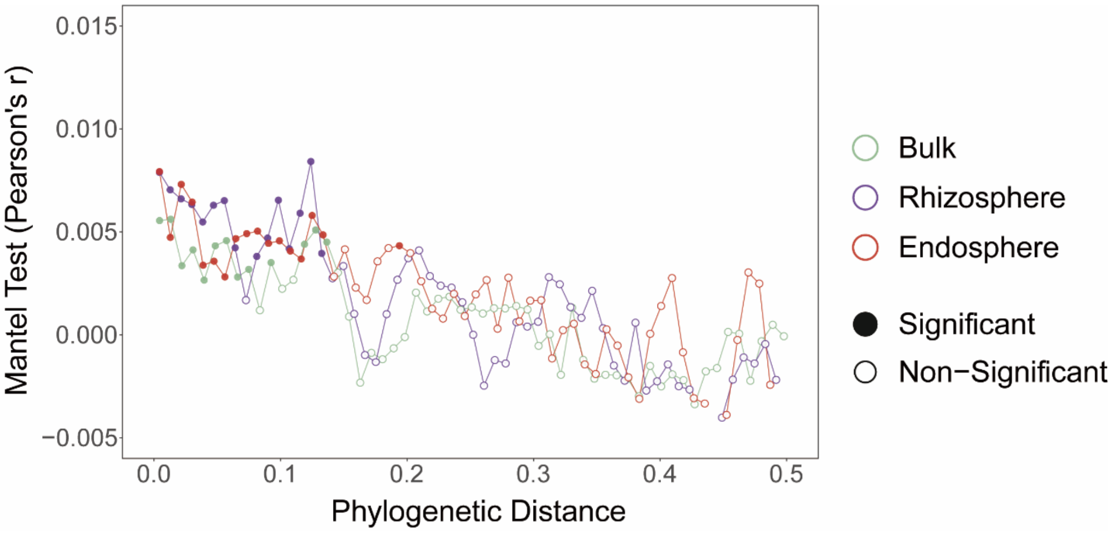

Figure S3 Relationship between the phylogenetic distance and niche difference for bioavailable arsenic concentrations based on Mantel Test. Color of circles represents different habitats: bulk soil (green), rhizosphere (purple) and endosphere (red). Solid and open symbols denote significant and non-significant correlations at $p<0.05$.

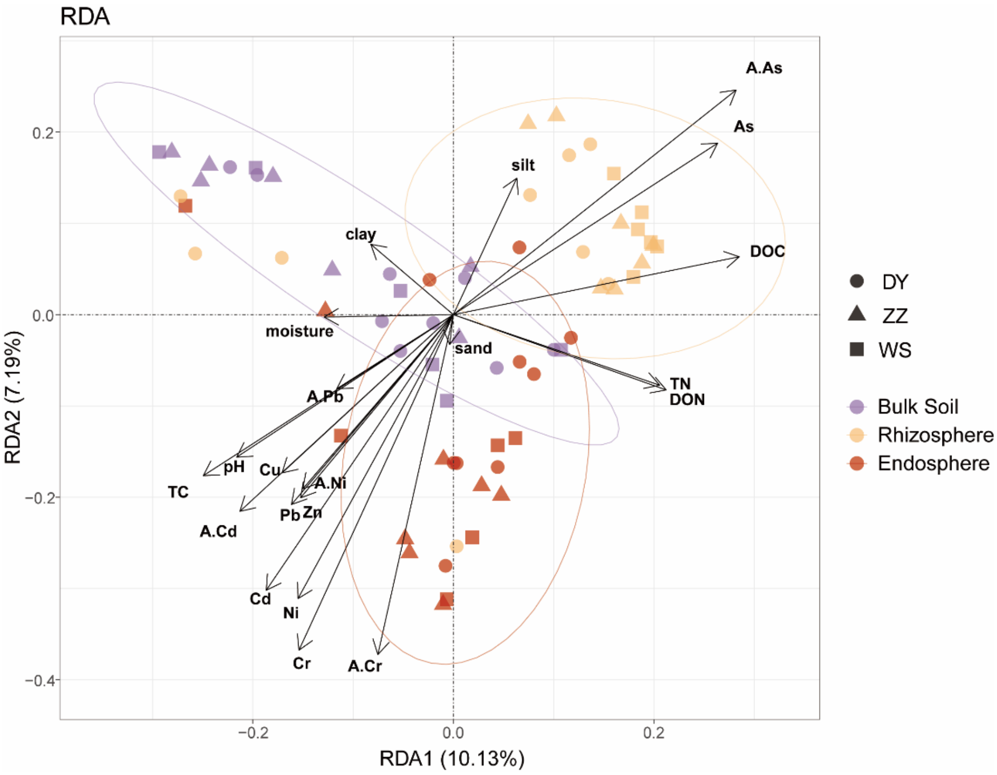

Figure S4 Redundancy Analysis (RDA) plot illustrating the relationships between soil properties and microbial community composition. The RDA1 and RDA2 axes represent the first and second dimensions of variability, explaining the predominant patterns in the dataset. Purple, yellow and red color denote microbial community from bulk soil, rhizosphere and endosphere, respectively. Circles, triangles and rectangles represent samples from DY, ZZ and WS sites, respectively. Arrows indicate the direction and magnitude of variables.

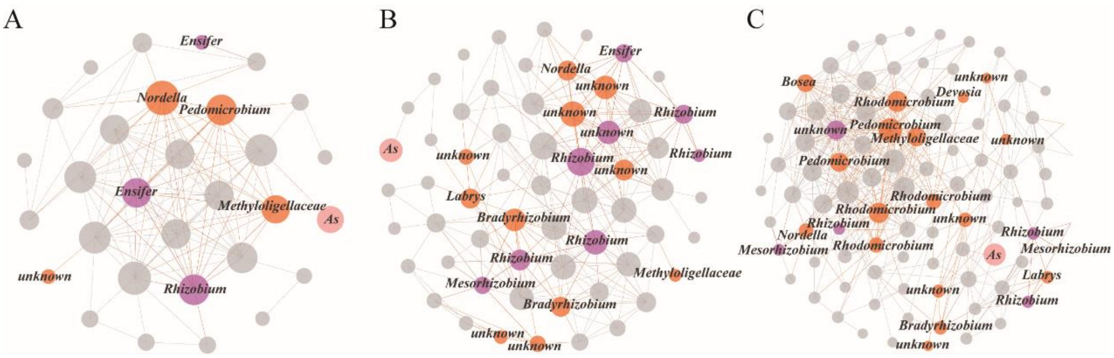

Figure S5 Bacterial sub-networks associated with arsenic in the (A) bulk soil, (B) rhizosphere, and (C) endosphere habitats as revealed by bacterial interactions within the arsenic-linked network module. All colored nodes depict taxa from Rhizobiales, with purple nodes highlighting bacteria from *Rhizobiaceae*.

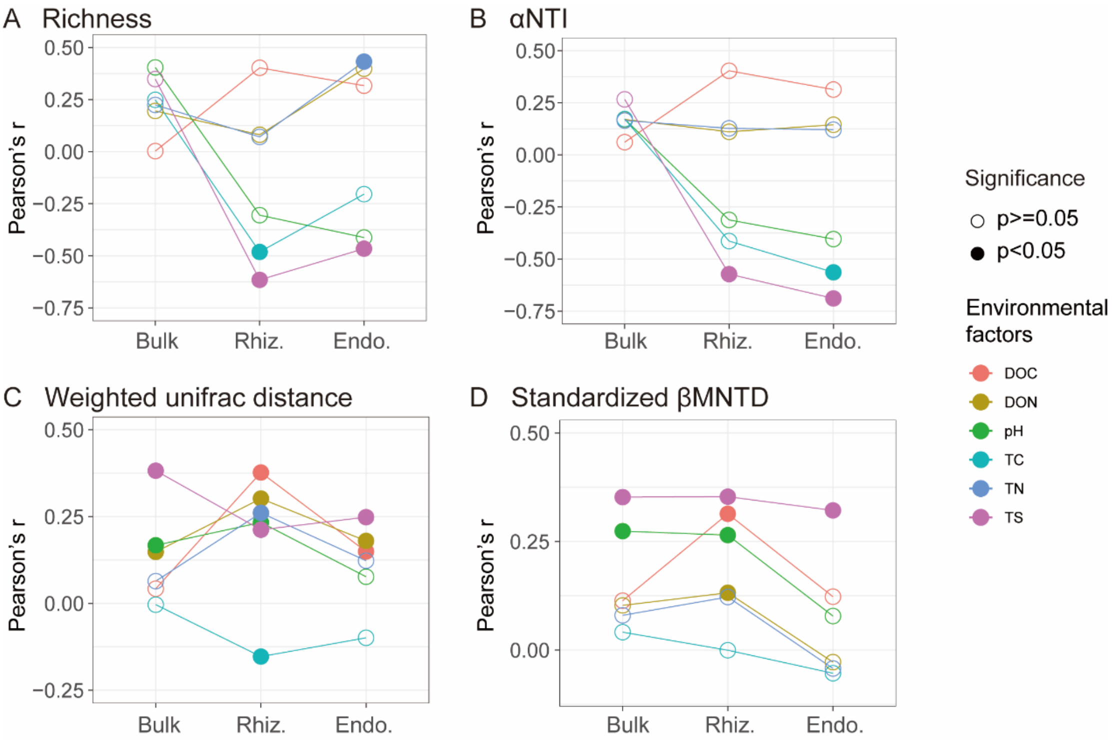

Figure S6 Correlations of bacterial richness (A), αNTI (B), weighted UniFrac dissimilarity (C) and βMNTD (D) with basic soil properties (pH, dissolved organic carbon (DOC), total carbon (TC), dissolved organic nitrogen (DON), total nitrogen (TN), and total sulfur (TS)) as a function of soil-root habitats. Solid and open symbols denote significant and non-significant correlations at $p \leq 0.05$.

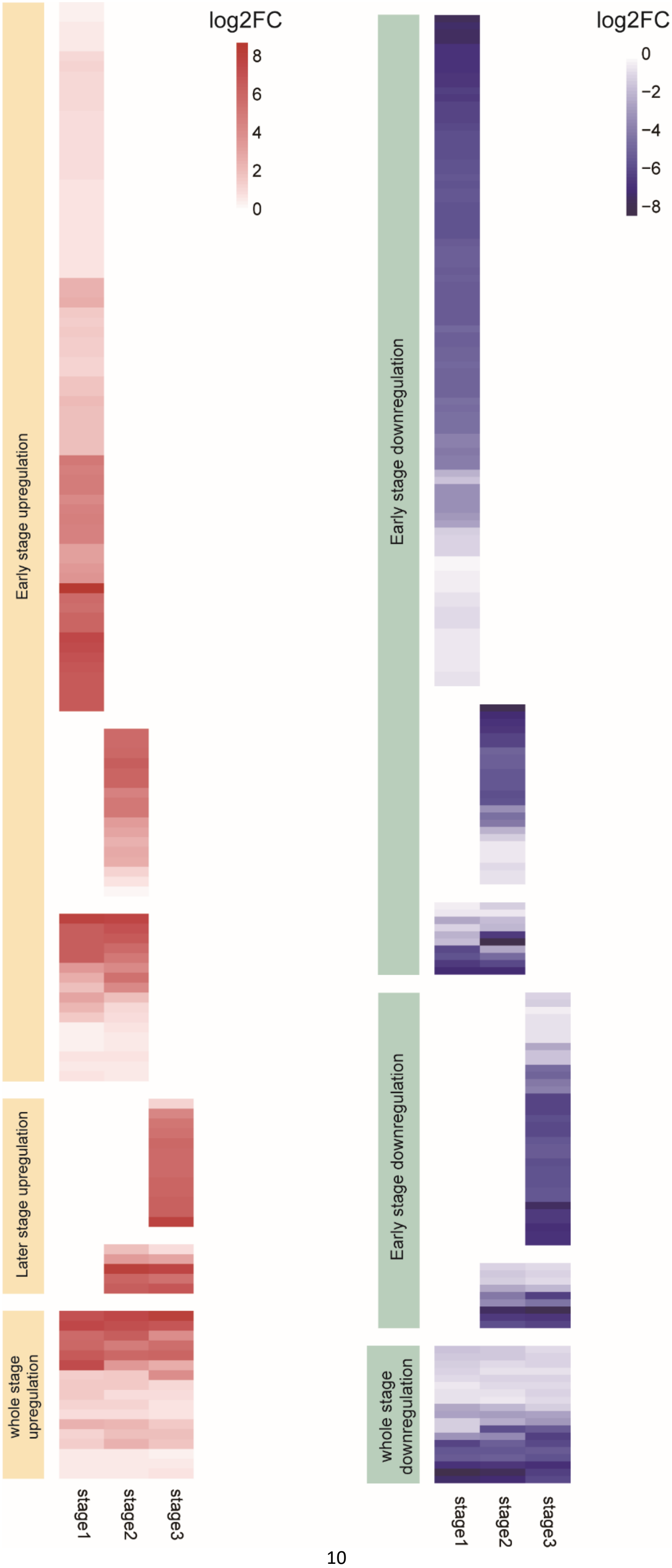

log2FC
8
6
4
2
0
Early stage upregulation
Later stage upregulation
whole stage upregulation
stage1
stage2
stage3
log2FC
0
−2
−4
−6
−8
Early stage downregulation
Early stage downregulation
whole stage downregulation
stage1
stage2
stage3

Figure S7 Heatmap displaying log2 fold-changes between control and As treatment at three growth stages of *Pteris vittata* at 10 (stage 1), 20 (stage 2), and 30 (stage 3) days. Upregulation under As-treatment tends toward darker red (left) while downregulation under As-treatment tends toward darker blue (right). Each row represents a differentially abundant ASV detected as significant (Wald test, $p<0.05$, FDR < 0.1) in at least one pair-wise comparison.

Table S1 Kruskal-Wallis Rank Sum Test to assess the difference of each environmental factors across ZZ, DY and WS site

Factor	p-value
pH	0.237
DOC	0.055
DON	0.345
TC	0.125
TN	0.178
clay	0.208
sand	0.283
silt	0.250

Table S2 The total heavy metal concentration in bulk soil

Sample	As	Cr	Ni	Cu	Zn	Cd	Pb
DY-1	2028	76.7	25.4	456	1238	56.7	1167
DY-2	565	54.7	21	225	454	17.7	193
DY-3	2598	66.5	27.7	344	1564	64.9	688
DY-4	947	67.4	21.8	147	420	13.9	245
DY-5	2732	76.3	24.8	430	1071	48.1	1100
DY-6	1738	75.5	26.4	345	950	55.9	735
DY-7	6971	89	26.9	1123	1284	96.3	2390
DY-8	3603	205	57.4	1115	2416	73.3	2994
DY-9	2640	99.1	37.2	1089	1256	61.2	1273
ZZ-1	363	206	125	525	33200	471	4895
ZZ-2	154	220	77.6	219	15000	158	3372
ZZ-3	1085	297	160	1217	40900	472	10270
ZZ-4	399	70.1	61.8	1319	134000	386	29170
ZZ-5	537	220	93.2	1093	38300	874	8689
ZZ-6	816	226	184	2007	50700	474	12320
ZZ-7	503	176	74.4	760	34600	258	10830
WS-1	18698	33.8	16	234	270	1.12	71.5
WS-2	2316	22.7	28	277	91.9	0.15	54.6
WS-3	9386	18.5	8.46	219	123	0.44	55.1
WS-4	3245	7.28	2.72	77.6	81.6	0.24	48.9
WS-5	30346	47.3	23	192	387	1.45	56.5
WS-6	8098	19.6	7.75	159	177	0.59	66.1

Note: Concentration is in mg/kg.

Table S3 The bioavailable heavy metal concentration in bulk soil

Sample	As	Cr	Ni	Cu	Zn	Cd	Pb
DY-1	98.098	0.299	2.733	95.935	670.629	34.138	91.546
DY-2	5.359	0.295	1.233	65.991	243.625	10.771	19.011
DY-3	290.744	0.284	4.271	88.995	1082.975	47.776	62.916
DY-4	18.025	0.383	0.848	27.378	207.514	8.844	69.132
DY-5	246.704	0.418	2.665	111.716	561.374	28.056	86.355
DY-6	122.951	0.073	1.075	11.155	187.144	23.537	5.013
DY-7	309.957	0.154	3.195	345.206	493.728	42.485	22.95
DY-8	103.216	0.925	6.903	255.113	1437.705	46.56	177.215
DY-9	45.18	0.255	5.491	265.723	549.249	32.212	36.436
ZZ-1	1.883	2.56	6.923	1.854	16032.264	141.166	294.72
ZZ-2	1.381	0.844	6.192	34.162	10036.232	129.687	1104.239
ZZ-3	6.252	4.255	18.49	3.094	16878.851	58.885	251.968
ZZ-4	0.485	0.181	6.393	113.482	56059.93	250.088	14408.981
ZZ-5	2.548	2.138	6.746	26.196	15855.912	634.156	2200.445
ZZ-6	1.954	1.147	25.96	169.647	21930.255	276.747	1140.822
ZZ-7	2.365	2.511	5.084	36.454	13590.701	161.96	1566.934
WS-1	535.957	0.053	0.585	9.044	124.505	0.249	0.071
WS-2	41.431	0.178	4.697	35.634	20.552	0.097	0.502
WS-3	282.482	0.089	0.406	21.876	19.972	0.125	0.12
WS-4	328.962	0.055	0.19	6.661	13.248	0.099	0.193
WS-5	461.801	0.041	0.674	8.829	146.841	0.301	0.028
WS-6	372.942	0.062	0.334	4.482	102.003	0.375	1.813

Note: Concentration is in mg/kg.

Table S4 Multiple linear regression results for microbial richness versus soil nutrient, soil texture, moisture and bioavailable heavy metal concentration in rhizosphere of *Pteris vittata*.

Model	t value	Pr(>|t|)	Significance
(Intercept)	2.977	0.0309	*
A.As	2.977	0.0309	*
TN	-1.634	0.1633	
TC	2.435	0.059	.
TS	-0.22	0.8348	
DOC	1.237	0.2709	
DON	1.101	0.3209	
pH	-0.237	0.8221	
A.Cr	-1.176	0.2924	
A.Ni	-0.813	0.453	
A.Cu	-1.886	0.118	
A.Zn	1.247	0.2676	
A.Cd	0.465	0.6614	
A.Pb	-1.442	0.2089	
clay	-1.007	0.36	
sand	-2.301	0.0697	.
silt	1.451	0.2064	
moisture	0.452	0.424	

Adjusted R-squared: 0.848

p-value: 0.014

Asterisks indicate the significance (*: p<0.05)

Table S5 Topological parameters of bacterial co-occurrence networks in the bulk soil, rhizosphere and endosphere habitats

Topological parameter	Bulk	Rhizosphere	Endosphere
Node number	360	229	238
Edge number	1098	624	620
Average degree	6.1	5.45	5.21
Network density	0.017	0.024	0.022
Average path length	3.862	4.806	3.046
Diameter	10	14	8

Table S6 Influence of experimental factors and their interaction on beta diversities of rhizosphere community

Habitat	Stage	Treatment	Stage × Treatment
$R^2 = 0.538$	$R^2 = 0.406$	$R^2 = 0.162$	$R^2 = 0.482$
P = 0.001	P = 0.001	P = 0.005	P = 0.001

Table S7 Differentially abundant ASVs in the core assembly module (CAM)

ASV_family	As exposure												Control											
	Stage 1 (Rhizosphere vs. Bulk soil)				Stage 2 (Rhizosphere vs. Bulk soil)				Stage 3 (Rhizosphere vs. Bulk soil)				Stage 1 (Rhizosphere vs. Bulk soil)				Stage 2 (Rhizosphere vs. Bulk soil)				Stage 3 (Rhizosphere vs. Bulk soil)			
	LFC	padj	conf_low	conf_high	LFC	padj	conf_low	conf_high	LFC	padj	conf_low	conf_high	LFC	padj	conf_low	conf_high	LFC	padj	conf_low	conf_high	LFC	padj	conf_low	conf_high
Vicinamibacteraceae	0.65	1.70E-05	0.40	0.91	0.72	4.72E-16	0.56	0.89	0.67	8.12E-10	0.47	0.87	0.53	2.62E-03	0.26	0.80	0.76	9.95E-11	0.55	0.98	0.75	1.83E-06	0.47	1.04
Gemmatimonadaceae	0.82	9.54E-05	0.47	1.17	0.92	1.30E-06	0.58	1.27	1.26	5.48E-07	0.81	1.71	0.40	4.10E-03	0.19	0.61	0.62	5.08E-04	0.31	0.92	1.16	8.05E-26	0.95	1.37
Gemmatimonadaceae	0.57	5.99E-06	0.36	0.79	0.40	5.79E-04	0.20	0.61	0.51	8.94E-03	0.18	0.84	0.48	6.15E-03	0.22	0.75	0.82	8.63E-09	0.56	1.07	0.64	5.06E-08	0.43	0.85
Gemmatimonadaceae	0.92	4.55E-07	0.60	1.23	1.08	6.28E-10	0.76	1.40	1.22	2.08E-08	0.83	1.62	0.49	2.64E-02	0.18	0.80	0.79	1.17E-05	0.47	1.10	1.26	9.45E-23	1.02	1.50
Comamonadaceae	12.08	1.68E-40	10.37	13.79	12.38	5.93E-45	10.72	14.05	11.43	9.89E-31	9.58	13.28	11.73	6.35E-40	10.06	13.40	11.89	3.26E-35	10.09	13.68	11.05	2.53E-32	9.30	12.80
Gemmatimonadaceae	0.57	7.26E-03	0.24	0.89	0.65	6.11E-04	0.32	0.98	0.95	4.36E-13	0.71	1.19	0.69	4.34E-05	0.41	0.97	0.98	2.61E-11	0.71	1.24	0.67	8.21E-07	0.43	0.91
uncultured	0.94	2.56E-04	0.51	1.37	0.77	2.42E-05	0.45	1.09	0.75	8.59E-07	0.47	1.02	0.69	3.59E-05	0.41	0.96	0.99	2.01E-12	0.73	1.25	0.51	6.31E-04	0.25	0.77
Longimicrobiaceae	10.25	3.43E-19	8.16	12.34	12.12	1.70E-11	8.81	15.42	11.24	1.70E-11	8.16	14.32	10.26	1.80E-18	8.11	12.40	11.46	4.09E-19	9.08	13.83	10.29	3.12E-07	6.68	13.91
Gemmatimonadaceae	1.08	3.88E-06	0.68	1.48	2.65	6.97E-33	2.23	3.06	3.09	3.25E-33	2.61	3.57	1.10	2.19E-04	0.62	1.59	2.20	2.58E-26	1.82	2.59	2.94	2.76E-22	2.38	3.50
Sphingomonadaceae	10.44	2.71E-26	8.62	12.26	10.35	3.06E-32	8.70	12.00	10.39	1.20E-27	8.61	12.18	10.21	4.17E-29	8.52	11.91	10.00	6.49E-27	8.27	11.73	10.76	3.05E-32	9.05	12.47
Comamonadaceae	4.34	8.77E-03	1.77	6.90	10.11	6.75E-18	7.94	12.29	9.66	6.43E-26	7.94	11.37	1.89	5.48E-05	1.12	2.66	3.38	4.72E-08	2.27	4.50	4.62	5.10E-03	1.83	7.42
Comamonadaceae	2.52	7.46E-03	1.05	3.98	5.57	8.72E-07	3.54	7.59	4.51	2.64E-04	2.34	6.67	1.05	4.96E-04	0.57	1.53	2.39	2.02E-12	1.77	3.02	3.09	4.94E-09	2.13	4.05
uncultured	0.64	2.34E-02	0.22	1.06	1.36	1.81E-11	0.99	1.73	1.38	1.59E-14	1.05	1.72	0.66	1.85E-02	0.26	1.06	1.01	6.26E-06	0.61	1.40	1.18	6.68E-11	0.85	1.51
Gemmataceae	9.11	6.80E-11	6.62	11.61	10.68	2.85E-16	8.26	13.10	9.15	5.09E-22	7.38	10.92	8.39	1.81E-06	5.40	11.37	10.11	1.39E-06	6.38	13.84	7.73	7.67E-08	5.13	10.32
Opitutaceae	10.01	3.80E-13	7.53	12.48	9.78	1.17E-24	8.00	11.56	9.76	5.68E-17	7.59	11.93	9.06	1.16E-21	7.31	10.80	10.27	2.86E-20	8.21	12.34	8.73	6.61E-18	6.86	10.60
Comamonadaceae	9.50	5.31E-19	7.55	11.45	10.25	1.69E-29	8.54	11.96	9.90	2.98E-25	8.12	11.69	7.99	1.62E-16	6.22	9.76	9.65	7.38E-26	7.94	11.36	9.02	1.08E-19	7.18	10.85
Comamonadaceae	3.87	1.97E-10	2.79	4.96	3.41	5.84E-15	2.60	4.22	4.03	4.39E-16	3.11	4.96	2.31	2.57E-04	1.29	3.33	5.99	1.86E-14	4.55	7.44	3.80	1.24E-12	2.81	4.78
Rhizobiaceae	8.81	1.34E-16	6.87	10.74	9.02	3.44E-23	7.33	10.72	8.85	2.37E-20	7.07	10.63	7.65	1.18E-13	5.79	9.51	8.79	1.64E-18	6.93	10.65	9.01	1.29E-21	7.25	10.76
Anaerolineaceae	8.58	6.62E-03	3.66	13.49	9.29	1.19E-19	7.39	11.19	9.52	1.83E-25	7.81	11.22	6.35	6.94E-04	3.37	9.33	8.99	3.51E-18	7.07	10.91	9.23	1.83E-16	7.16	11.30
uncultured	9.30	5.54E-11	6.77	11.83	9.60	5.22E-23	7.79	11.41	9.15	2.63E-17	7.14	11.17	8.14	4.19E-15	6.26	10.02	9.18	2.33E-20	7.34	11.03	7.86	3.23E-13	5.88	9.85
Phaselicystidaceae	7.79	3.48E-06	4.91	10.66	9.02	2.63E-19	7.16	10.88	9.19	3.59E-20	7.33	11.05	6.78	3.49E-09	4.76	8.80	9.21	5.16E-20	7.35	11.08	8.92	1.47E-19	7.09	10.75
Microscillaceae	8.04	2.38E-13	6.07	10.01	8.60	2.22E-16	6.66	10.54	8.76	4.43E-15	6.69	10.83	6.99	3.52E-08	4.78	9.20	8.72	2.66E-18	6.87	10.58	8.91	1.32E-18	7.03	10.78
Diplorickettsiaceae	8.41	4.22E-13	6.33	10.50	8.27	1.34E-17	6.48	10.06	8.07	4.43E-15	6.16	9.97	8.26	5.14E-18	6.51	10.00	8.67	4.09E-19	6.87	10.47	8.05	1.92E-15	6.18	9.92
Solimonadaceae	6.56	5.60E-03	2.86	10.25	9.11	2.45E-06	5.67	12.56	9.09	2.18E-12	6.70	11.48	6.87	3.59E-05	4.13	9.62	9.59	3.56E-22	7.76	11.43	7.60	8.05E-12	5.56	9.65
BIrii41	5.52	1.02E-03	2.80	8.24	5.80	7.58E-09	3.98	7.63	8.40	6.02E-16	6.47	10.32	4.21	8.79E-04	2.21	6.22	4.57	7.00E-07	2.93	6.22	4.82	3.38E-03	2.03	7.62
Chitinophagaceae	7.69	4.74E-12	5.70	9.68	8.66	1.42E-06	5.46	11.87	8.29	4.56E-16	6.40	10.19	7.63	2.29E-13	5.76	9.51	8.63	8.91E-18	6.77	10.50	7.89	7.76E-08	5.24	10.54
Spirochaetaceae	7.98	5.54E-11	5.81	10.15	8.36	1.64E-15	6.41	10.30	8.79	8.27E-22	7.08	10.50	7.42	2.67E-11	5.43	9.40	8.34	5.28E-17	6.50	10.19	7.54	9.94E-14	5.68	9.41
Microscillaceae	8.31	4.79E-05	4.86	11.76	7.24	7.64E-07	4.62	9.86	7.89	5.81E-09	5.42	10.37	6.44	9.02E-03	2.78	10.11	7.43	3.81E-07	4.81	10.04	7.72	5.98E-09	5.30	10.13
Reyranellaceae	8.19	4.47E-11	5.97	10.40	5.38	9.93E-08	3.56	7.20	8.01	4.04E-12	5.88	10.14	6.77	4.18E-08	4.62	8.93	7.84	1.73E-07	5.15	10.52	7.69	9.73E-07	4.88	10.51
Hydrogenedensaceae	7.38	1.02E-08	5.11	9.65	8.36	1.77E-15	6.41	10.31	8.43	6.02E-16	6.49	10.37	5.65	2.87E-05	3.42	7.88	8.33	5.16E-19	6.60	10.07	8.04	2.10E-14	6.10	9.98
Microscillaceae	7.38	1.78E-08	5.07	9.68	8.11	5.77E-11	5.84	10.38	7.54	9.38E-12	5.50	9.58	6.87	1.75E-09	4.86	8.88	8.38	7.99E-18	6.57	10.18	7.91	1.68E-11	5.75	10.07
Anaerolineaceae	7.32	9.90E-06	4.50	10.14	7.96	3.54E-12	5.85	10.07	8.56	1.42E-16	6.63	10.48	6.63	3.87E-04	3.64	9.62	7.82	6.38E-14	5.90	9.74	7.08	9.34E-07	4.49	9.66
Amb-16S-1323	7.34	2.81E-06	4.65	10.03	7.21	9.46E-07	4.58	9.84	7.45	1.25E-12	5.51	9.39	6.78	3.49E-09	4.76	8.80	7.46	5.43E-13	5.56	9.36	7.23	1.13E-09	5.07	9.40
Azospirillales_Incertae_Sedis	7.61	7.40E-10	5.41	9.82	8.04	3.39E-13	6.00	10.07	7.47	1.30E-10	5.34	9.61	6.13	5.41E-07	4.04	8.22	7.55	1.56E-11	5.50	9.60	7.19	3.56E-10	5.10	9.29
Sphingomonadaceae	7.51	1.58E-11	5.52	9.49	7.50	3.32E-13	5.60	9.39	6.37	2.26E-03	2.78	9.96	6.52	6.23E-08	4.43	8.61	7.38	1.71E-10	5.27	9.48	7.10	1.30E-10	5.08	9.13
SBR1031	6.66	6.62E-03	2.84	10.48	8.05	3.86E-03	3.31	12.78	7.57	3.84E-07	4.88	10.26	5.77	2.24E-03	2.85	8.69	7.76	3.55E-14	5.87	9.65	6.82	1.04E-09	4.78	8.86
Obscuribacteraceae	6.23	1.16E-02	2.43	10.03	7.53	3.84E-09	5.20	9.85	8.23	1.55E-11	5.98	10.48	5.97	2.28E-03	2.94	9.00	7.67	3.29E-14	5.81	9.53	7.02	5.46E-06	4.28	9.77
Hyphomonadaceae	7.01	1.78E-08	4.82	9.20	7.44	8.81E-11	5.34	9.55	6.76	1.41E-08	4.59	8.93	6.60	8.89E-05	3.85	9.34	7.49	5.48E-13	5.58	9.40	6.45	1.16E-08	4.40	8.51
uncultured	6.34	8.63E-03	2.60	10.07	7.37	6.04E-07	4.72	10.01	7.25	2.35E-10	5.14	9.35	5.62	4.54E-02	1.78	9.45	7.42	2.10E-12	5.48	9.36	7.54	1.08E-11	5.51	9.58
TRA3-20	6.17	1.25E-02	2.38	9.97	6.66	1.08E-03	3.16	10.16	7.35	1.64E-10	5.24	9.46	6.43	4.37E-04	3.50	9.35	7.16	2.74E-06	4.45	9.87	6.83	1.23E-09	4.78	8.88
A4b	6.87	7.29E-05	3.95	9.78	6.01	4.08E-03	2.45	9.56	6.59	4.36E-07	4.24	8.94	6.92	1.07E-04	4.01	9.83	6.45	9.63E-05	3.57	9.33	5.73	2.03E-05	3.36	8.10
uncultured	6.65	2.18E-06	4.24	9.05	6.85	3.05E-08	4.61	9.09	6.15	1.82E-07	4.02	8.28	5.51	1.13E-04	3.18	7.84	7.66	2.01E-12	5.66	9.66	6.03	1.18E-04	3.29	8.76

LFC: Log2 Fold Change
padj: p-value adjusted by Benjamini-Hochberg method.
Conf_low: Lower confidence limit at the 95% confidence level.
Conf_high: Upper confidence limit at the 95% confidence level.

Table S8 Differentially abundant ASVs in the whole stage upregulated module (WSUM)

ASV_family	Rhizosphere											
	Stage 1 (As exposure vs. Bulk soil)				Stage 2 (As exposure vs. Bulk soil)				Stage 3 (As exposure vs. Bulk soil)			
	LFC	padj	conf_low	conf_high	LFC	padj	conf_low	conf_high	LFC	padj	conf_low	conf_high
Gemmatimonadaceae	0.62	2.47E-02	0.23	1.02	0.65	6.44E-03	0.30	1.00	0.69	6.22E-02	0.22	1.15
Bryobacteraceae	1.46	7.39E-08	1.00	1.93	1.66	2.93E-06	1.06	2.25	4.05	3.70E-03	2.03	6.08
BSV26	1.29	1.48E-04	0.74	1.85	2.04	4.87E-02	0.68	3.41	2.06	4.09E-02	0.75	3.38
Caloramatoraceae	6.07	1.22E-03	3.15	9.00	5.01	9.92E-02	1.26	8.76	5.69	5.97E-02	1.86	9.53
Microscillaceae	6.83	5.86E-08	4.68	8.99	6.06	2.51E-02	2.35	9.77	6.21	2.51E-03	3.20	9.22
uncultured	1.28	1.60E-02	0.51	2.05	1.24	1.70E-05	0.77	1.71	0.88	2.84E-02	0.34	1.41
Opitutaceae	1.67	8.77E-03	0.72	2.62	0.93	4.67E-02	0.31	1.54	0.98	3.84E-02	0.36	1.60
Peptostreptococcaceae	5.89	3.36E-06	3.75	8.02	6.56	1.96E-08	4.54	8.59	4.10	6.39E-03	1.94	6.25
Blastocatellaceae	7.40	1.79E-06	4.78	10.03	3.53	6.03E-02	1.11	5.95	2.76	3.64E-02	1.03	4.49
Intrasporangiaceae	0.99	8.86E-08	0.67	1.31	0.90	1.72E-03	0.46	1.33	0.79	6.56E-03	0.37	1.21
Pseudomonadaceae	7.16	1.12E-03	3.74	10.59	7.69	6.78E-12	5.70	9.67	8.10	1.22E-08	5.69	10.52
Gemmatimonadaceae	1.43	1.01E-06	0.93	1.92	2.59	8.06E-04	1.40	3.79	1.93	8.01E-02	0.56	3.29
Microscillaceae	2.53	5.11E-02	0.75	4.31	2.41	5.76E-05	1.44	3.38	1.69	2.02E-03	0.89	2.49
Nitrosomonadaceae	7.67	7.39E-08	5.22	10.12	7.35	1.47E-12	5.51	9.20	6.98	4.79E-05	4.28	9.68
Xanthomonadaceae	1.75	8.86E-03	0.75	2.74	1.74	4.69E-03	0.83	2.65	1.23	5.92E-03	0.59	1.87
Gemmatimonadaceae	0.66	1.15E-02	0.27	1.04	0.67	4.69E-03	0.32	1.02	0.71	8.38E-02	0.20	1.22
Gemmatimonadaceae	0.63	6.14E-04	0.34	0.92	0.67	1.20E-02	0.29	1.04	0.49	5.60E-02	0.16	0.82

LFC: Log2 Fold Change

padj: p-value adjusted by Benjamini-Hochberg method.

Conf_low: Lower confidence limit at the 95% confidence level.

Conf_high: Upper confidence limit at the 95% confidence level.
